# A Machine Learning-Based Approach to Predict Prognosis and Length of Hospital Stay in Adults and Children With Traumatic Brain Injury: Retrospective Cohort Study

**DOI:** 10.2196/41819

**Published:** 2022-12-09

**Authors:** Cheng Fang, Yifeng Pan, Luotong Zhao, Zhaoyi Niu, Qingguo Guo, Bing Zhao

**Affiliations:** 1 Department of Neurosurgery The Second Affiliated Hospital of Anhui Medical University Anhui Medical University Hefei China; 2 The School of Big Data and Artificial Intelligence Anhui Xinhua University Hefei China

**Keywords:** convolutional neural network, machine learning, neurosurgery, support vector machine, support vector regression, traumatic brain injury

## Abstract

**Background:**

The treatment and care of adults and children with traumatic brain injury (TBI) constitute an intractable global health problem. Predicting the prognosis and length of hospital stay of patients with TBI may improve therapeutic effects and significantly reduce societal health care burden. Applying novel machine learning methods to the field of TBI may be valuable for determining the prognosis and cost-effectiveness of clinical treatment.

**Objective:**

We aimed to combine multiple machine learning approaches to build hybrid models for predicting the prognosis and length of hospital stay for adults and children with TBI.

**Methods:**

We collected relevant clinical information from patients treated at the Neurosurgery Center of the Second Affiliated Hospital of Anhui Medical University between May 2017 and May 2022, of which 80% was used for training the model and 20% for testing via screening and data splitting. We trained and tested the machine learning models using 5 cross-validations to avoid overfitting. In the machine learning models, 11 types of independent variables were used as input variables and Glasgow Outcome Scale score, used to evaluate patients’ prognosis, and patient length of stay were used as output variables. Once the models were trained, we obtained and compared the errors of each machine learning model from 5 rounds of cross-validation to select the best predictive model. The model was then externally tested using clinical data of patients treated at the First Affiliated Hospital of Anhui Medical University from June 2021 to February 2022.

**Results:**

The final convolutional neural network–support vector machine (CNN-SVM) model predicted Glasgow Outcome Scale score with an accuracy of 93% and 93.69% in the test and external validation sets, respectively, and an area under the curve of 94.68% and 94.32% in the test and external validation sets, respectively. The mean absolute percentage error of the final built convolutional neural network–support vector regression (CNN-SVR) model predicting inpatient time in the test set and external validation set was 10.72% and 10.44%, respectively. The coefficient of determination (*R*^2^) was 0.93 and 0.92 in the test set and external validation set, respectively. Compared with back-propagation neural network, CNN, and SVM models built separately, our hybrid model was identified to be optimal and had high confidence.

**Conclusions:**

This study demonstrates the clinical utility of 2 hybrid models built by combining multiple machine learning approaches to accurately predict the prognosis and length of stay in hospital for adults and children with TBI. Application of these models may reduce the burden on physicians when assessing TBI and assist clinicians in the medical decision-making process.

## Introduction

### Background

More than 50 million people worldwide suffer from traumatic brain injury (TBI) each year, which reduces patient quality of life and leads to high morbidity and mortality. Approximately half of the global population is likely to experience one or more brain injuries in their lifetime [1,2]. The greatest burden of TBI has been reported in low- and middle-income countries [3], where medical resources are limited and medical experience is lacking and patients often have a poor prognosis, further adding to the medical burden on society. Therefore, creating a tool that can predict patient prognosis and length of stay to aid clinician medical decisions is essential to achieve precision medicine [4].

With the popularity of computers and the rapid development of computer science, people are increasingly using computer knowledge to solve practical problems, and machine learning methods are gaining more and more attention from scientists. Machine learning is a scientific discipline that focuses on how computers learn from data and has been widely used in military and civilian applications [5]. Research incorporating computer algorithms into medicine has also been reported [6-10]. Currently, common machine learning methods include artificial neural networks and back propagation (BP) neural networks, some of the classical algorithms that have been widely used, but their drawback of easy overfitting is difficult to solve. Novel algorithms such as convolutional neural networks (CNN), support vector machine (SVM), and support vector regression (SVR) have solved this drawback well, allowing for more accurate machine learning models to be built. There has been very little research into the integration of these algorithms into clinical practice, let alone into complex studies such as predicting patient prognosis and length of stay. CNNs are a class of feedforward neural networks that incorporate convolutional computation, have deep structure, and are one of the representative algorithms for deep learning [11]. CNNs are built to mimic biological visual perception mechanisms and can directly process 2D images, hence their wide application in image processing [12,13]. Considering that CNN has achieved great success in the image field, we would like to see if CNN can also have good prediction and classification results when the input data is structured data. SVM is a class of generalized linear classifier that performs binary classification of data in a supervised learning fashion, where the decision boundary is a maximum-margin hyperplane solved for the learned samples. While SVM itself is proposed for classification problems, SVR is an important application branch of SVM, which is an application of SVM for regression prediction problems, both of which are applicable to our study. Compared with traditional machine learning methods, CNN, SVM, and SVR have faster learning speed, better network generalization, and more accurate classification and prediction of variables.

### Aim

The aim of this study is to apply the latest algorithms in machine learning to medicine for outcome prediction based on relevant clinical data. Machine learning methods create a mapping relationship between input and output data through the analytical processing of raw data and the application of various prediction algorithms. CNN, SVM, and SVR are among the representative new generation algorithms. Therefore, we combine the advantages of these methods and build two hybrid models for patient prediction. In this study, we aim to demonstrate that both methods are effective in predicting patients with traumatic injuries, and we hope to provide inspiration to future researchers working in health care information analysis.

## Methods

### Ethics Approval

This retrospective cohort study was approved by the Ethics Committee of the Second Affiliated Hospital of Anhui Medical University in December of 2020 (approval number S20210098). Participants or proxies signed the relevant informed consent forms within 24 hours of admission.

### Participants

The predictive model was developed based on relevant data from 1001 patients registered at the Neurosurgery Center of the Second Affiliated Hospital of Anhui Medical University with traumatic craniocerebral injury between May 2017 and May 2022 and at the First Hospital of Anhui Medical University between June 2021 and February 2022. By random splitting, we used 80% of the data in the training model and the remaining 20% to test model performance. We also collected clinical data from 111 patients at the First Affiliated Hospital of Anhui Medical University as a test cohort for external validation of the model.

**Figure 1:**
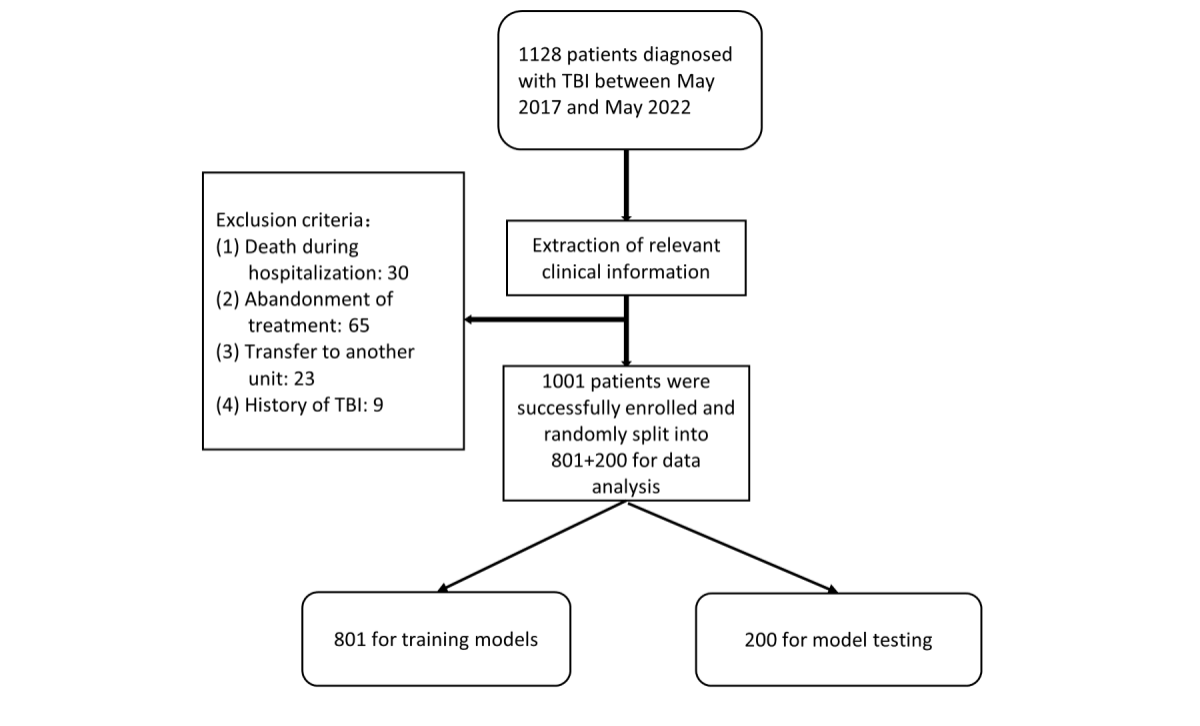
Training set and test set. TBI: traumatic brain injury.

**Figure 2:**
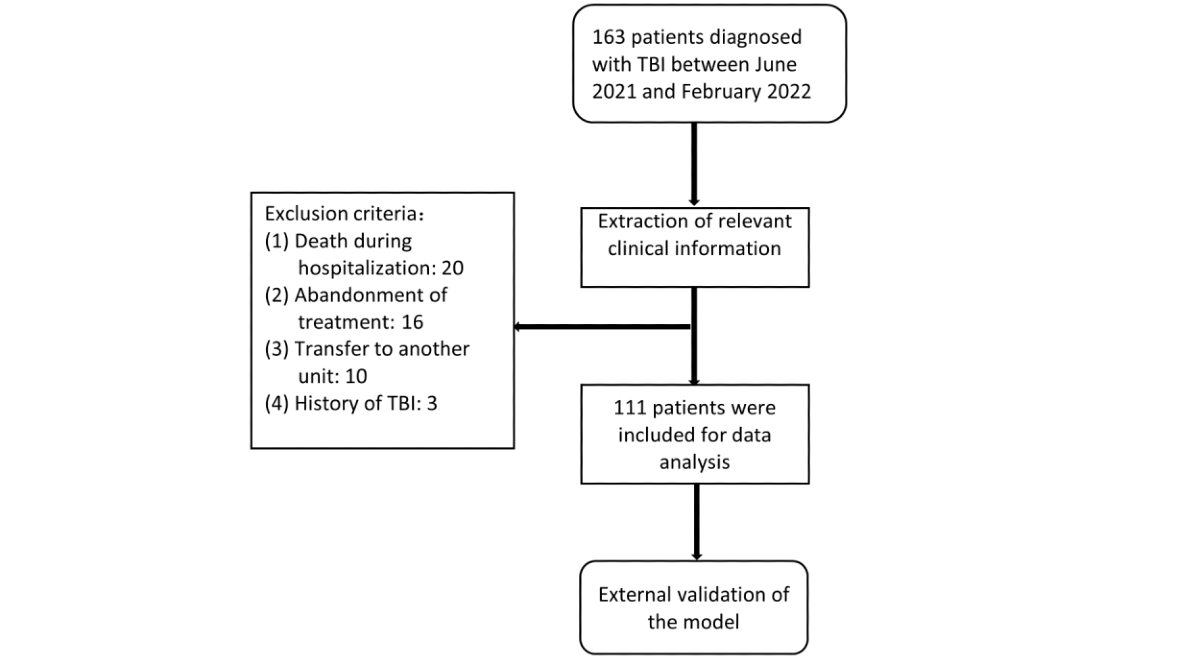
External validation set. TBI: traumatic brain injury.

We included all patients with complete demographic, clinical, and radiological data during this period, with inclusion criteria of (1) craniocerebral trauma as a result of external forces; (2) clinical diagnosis of craniocerebral trauma; and (3) complete clinical data including cases, course records, imaging examinations, and test reports were available. Our model prediction results included the length of stay of the patient; the decision regarding hospital discharge requires discussion by the treatment team and assessment by experienced neurosurgeons. Hence, the length of stay results were inaccurate when a patient died, when treatment was abandoned by the subjective will of the patient’s family, or when follow-up was lost due to transfer to other departments. Exclusion criteria were as follows: (1) patients died during hospitalization, (2) patients’ family requested to abandon treatment (including financial factors) and patients were automatically discharged, (3) patients with other severe injuries who required transfer to relevant departments for further treatment, and (4) patients with a history of craniocerebral injury (Figures 1 and 2).

### Data Collection

By reviewing relevant papers, formally trained neurologists extracted the necessary data for modeling from the electronical medical records of enrolled patients, including patients’ general characteristics (sex, age, and previous medical history), clinical and imaging data of patients with TBI (mechanism of TBI, loss of consciousness after injury, Glasgow Coma Scale [GCS] score, cranial computed tomography [CT] findings as jointly diagnosed by radiologists and neurosurgeons, other site injuries, treatment, admission to intensive care unit [ICU], and complications), and length of stay. The classification and definition of variables used to construct the predictive model are listed in Table 1.

**Table 1:** Variables used to construct the model.

Variable			Total (n=801), n (%)		Data type
**Age (years)**					
	≥17	49 (6.1)		Floating point data	
	18-44	220 (27.5)		Floating point data	
	45-64	321 (40.1)		Floating point data	
	65-74	147 (18.3)		Floating point data	
	≤75	64 (8.0)		Floating point data	
**Gender**					
	Male	577 (72.0)		Binary data	
	Female	224 (28.0)		Binary data	
**Past medical history**					
	Hypertension	146 (18.2)		Binary data	
	Diabetes	42 (5.2)		Binary data	
	Coronary artery disease	16 (2.0)		Binary data	
	Chronic renal failure	6 (0.7)		Binary data	
	Cerebral infarction	18 (2.2)		Binary data	
	Respiratory disorders	10 (1.2)		Binary data	
**Mechanism of injury**					
	Fall on the same plane	214 (26.7)		Binary data	
	Fall from high place	140 (17.5)		Binary data	
	Road accident	415 (51.8)		Binary data	
	Object striking the head	32 (4.0)		Binary data	
**Loss of consciousness**					
	Yes	385 (48.0)		Binary data	
	No	416 (52.0)		Binary data	
**Glasgow Coma Scale score**					
	13-15	480 (59.9)		Binary data	
	9-12	123 (15.4)		Binary data	
	3-8	198 (24.7)		Binary data	
**Neuroimaging results**					
	Epidural hematoma	244 (30.5)		Binary data	
	Subdural hematoma	434 (54.2)		Binary data	
	Subarachnoid hemorrhage	411 (51.3)		Binary data	
	Skull fracture	509 (63.5)		Binary data	
	Diffuse axonal injury	13 (1.6)		Binary data	
	Brain herniation	20 (2.5)		Binary data	
**Treatment**					
	Conservative	180 (22.5)		Binary data	
	Neurological surgery	621 (77.5)		Binary data	
**Other site injuries**					
	Fractures in other areas	232 (29.0)		Binary data	
	Visceral contusions	18 (2.2)		Binary data	
	Traumatic wet lung	103 (12.9)		Binary data	
	Pneumothorax	16 (2.0)		Binary data	
**Duration of intensive care unit stay (days)**					
	≤5	103 (12.9)		Floating point data	
	6-15	94 (11.7)		Floating point data	
	≥16	30 (3.7)		Floating point data	
**Complications**					
	Infections	191 (23.8)		Binary data	
	Tracheotomy	133 (16.6)		Binary data	
	Electrolyte disorders	230 (28.7)		Binary data	
	Impaired organ function	256 (32.0)		Binary data	
	Anemia	118 (14.7)		Binary data	
	Abnormal blood clotting	36 (4.5)		Binary data	
	Cerebrospinal fluid leakage	18 (2.2)		Binary data	
**Glasgow Outcome Scale score**					
	1	0(0)		Binary data	
	2	85 (10.6)		Binary data	
	3	97 (12.1)		Binary data	
	4	419 (52.3)		Binary data	
	5	200 (25.0)		Binary data	
**Length of stay in hospital (days)**					
	≤10	225 (28.1)		Floating point data	
	11-20	365 (45.6)		Floating point data	
	21-30	152 (19.0)		Floating point data	
	31-40	53 (6.6)		Floating point data	
	≥41	6 (0.7)		Floating point data	

The Glasgow Outcome Scale (GOS) score published by Jennett and Bond in 1975 [14] has emerged as one of the most widely used prognostic tools for assessing recovery after disability and TBI worldwide (Table 2). Patients with scores of 1 who died were excluded, those with scores of 5 and 4 were considered to have recovered, and patients with scores of 2 and 3 were considered to have a poorer prognosis. This supports our use of this tool as a criterion to evaluate patient prognosis.

**Table 2:** Descriptions of the categories of the Glasgow Outcome Scale.

Score		
1	Dead	As a direct result of brain trauma, or...due to secondary complications or other complications
2	Vegetative state	Patients who remain unresponsive and speechless…
3	Severe disability	Patient is conscious but needs the assistance of another person for some activities of daily living every day...
4	Moderate disability	Patient can look after themself at home, get out and about to the shops, and travel by public transport. However, some previous activities, either at work or in social life, are now no longer possible by reason of either physical or mental deficit...
5	Good recovery	Patient has the capacity to resume normal occupational and social activities, although there may be minor physical or mental deficits...social outcome should be included in the assessment here, such as leisure activities and family relationships

### Modeling

Neurologists were involved throughout the model development process and supervised the clinical application of the algorithm to ensure that the model’s predictions are meaningful and the research process meets the requirements of the ethics committee.

#### CNN-SVM Hybrid Model for Predicting GOS Score

The ability of CNN to extract data features is applicable to the processing of multidimensional input data in this study [15]. SVM can automatically identify support vectors that have better differentiation ability for classification. The resulting classifier can maximize the class-to-class interval, thus demonstrating better adaptability and higher classification accuracy [16], which is applicable to the prediction of GOS classification results. Therefore, this study combined CNN and SVM to build a hybrid CNN-SVM model for predicting the prognosis of patients with TBI. This model combined the respective advantages of CNN and SVM to improve model prediction accuracy and therefore exhibited greater advantages [17].

In the CNN-SVM hybrid model, the input layer consisted of 11 classes of input parameters, and the output layer was divided into 5 classes of GOS scores. Of the original 1001 data sets, 80% were randomly selected as the training set and 20% as the test set. Five rounds of model learning and validation were performed, and the average GOS classification accuracy of the 5 training and testing sessions was finally obtained. We developed the CNN-SVM hybrid model using the Pyrorch framework and Python 3.9 programming language.

**Figure 3:**
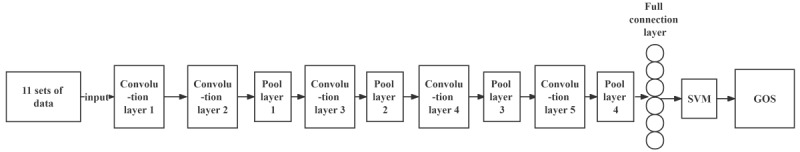
Convolutional neural network–support vector machine hybrid model building process. SVM: support vector machine; GOS: Glasgow Outcome Score.

The CNN-SVM model was used to make classification predictions for GOS score. Cross entropy was selected as the loss function of the model. Hyperparameters were selected through training, and the full training data were then retrained with the optimal parameters of the optimal model. After several attempts, rectified linear unit was selected as the activation function of the model [18]. The optimizer used momentum gradient descent [19]. The learning rate was set to 10-3, and the batch size was set to 64 according to the number of samples in the training set to ensure memory utilization and enhance processing speed for the same amount of data. The SVM model used an radical basis function kernel to avoid falling into local optimal solution. Penalty factor (*P*=100) and kernel parameter (γ=0.02) of the SVM were finalized using the grid search method [20]. The process of building the CNN-SVM hybrid model is shown in Figure 3; regarding the setup in CNN, details about our convolutional and pooling layers for structured data are shown in the Table 3.

**Table 3:** Parameter setting of CNN.

Network layer	Model parameter setting
Input layer	Data matrix
Convolution layer 1	64 1×1 convolution kernels; kernel_size = 5
Convolution layer 2	128 1×1 convolution kernels; kernel_size = 5
Pool layer 1	MaxPool; kernel_size =1; stride = 2
Convolution layer 3	128 1×1 convolution kernels; kernel_size = 5
Pool layer 2	MaxPool; kernel_size =1; stride = 2
Convolution layer 4	256 1×1 convolution kernels; kernel_size = 5
Pool layer 3	MaxPool; kernel_size =1; stride = 2
Convolution layer 5	516 1×1 convolution kernels; kernel_size = 5
Pool layer 4 (adaptive pooling layer)	Output 1D vector
Full connection layer	Output

#### CNN-SVR Hybrid Model to Predict Length of Stay

SVMs are used for classification problems. SVR is a key application branch of SVMs. SVR and SVM are distinct in that SVM aims to maximize the distance to the nearest sample point in the hyperplane, while SVR aims to minimize the distance to the farthest sample point in the hyperplane. Therefore, SVR is applicable to the regression prediction of length of stay in this study but not to the prediction of classification problems. We combined CNN and SVR to build a hybrid CNN-SVR model for predicting the hospital stay of patients with TBI. The input layer consisted of 11 input parameters, and the output layer was length of stay. We randomly selected 80% of the original 1001 data sets as the training set and 20% as the test set. In this study, the mean absolute percentage error (MAPE) was used to measure the error between the real hospitalization time and predicted hospitalization time in the model, as shown in equation (1), where *y_i_* represents the real data and *ŷ_i_* represents the predicted data. We developed the CNN-SVR hybrid model using the Pyrorch framework and Python 3.9 programming language.

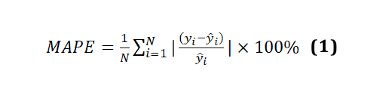



**Figure 4:**
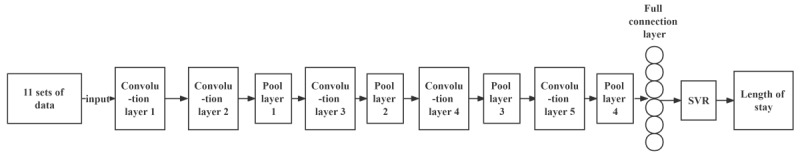
Convolutional neural network–support vector regression hybrid model building process. SVR: support vector regression.

The CNN-SVR model was used to predict the length of stay of patients with TBI. The mean square error was selected as the loss function for the regression prediction model. Hyperparameters were selected through training, and the full training data were retrained with the optimal parameters of the optimal model. After several attempts, rectified linear unit was selected as the activation function of the model. The optimizer was used as reported by Kingma and Ba [21]. The learning rate was set to 10-3, and the batch size was set to 32 according to the number of samples in the training set. The radical basis function kernel was used for the SVR model. The penalty factor of *P*=100 for SVR was finally determined using a grid search method with the kernel parameter γ=0.01. The process of building the CNN-SVR hybrid model shows in Figure 4, and details about our convolutional and pooling layers for structured data are shown in the Table 3.

## Results

### Evaluation Indicators

All 1001 valid samples were divided into training and test sets. The data were divided according to the rule of having similar statistical characteristics. The training set was divided into 0.8 of the total sample size and included the cross-validation data. Training and testing were repeated 5 times and averaged. For the classification prediction of GOS scores, the metric of precision was used to measure classification accuracy. For the prediction model of length of stay in patients with TBI, coefficient of determination (*R*^2^) and MAPE were used to examine model performance.

### Predicting the Prognosis of Patients With TBI: GOS Scores

To establish the optimal GOS classification model, this study compared the CNN-SVM model design with the construction of CNN, SVM, and BP neural network models. Accuracy indicates the proportion of correctly classified samples in the test set to the total sample size and can evaluate the predictive accuracy of the model, receiver operating characteristic curve is a composite indicator of sensitivity and specificity continuous variables, and area under the curve (AUC) can evaluate the generalization ability of the model. We used the accuracy and AUC to evaluate the advantages and disadvantages of the classification models. The accuracy and AUC of the 4 models (BP, CNN, SVM, and CNN-SVM) in the test data set are presented in Table 4. The classification results of the CNN-SVM hybrid model exhibited the highest accuracy and AUC; accuracy was 16.50%, 9.00%, and 5.50% higher than the BP, CNN, and SVM models, respectively, and AUC was 15.75%, 10.47%, and 6.33% higher than the BP, CNN, and SVM models, respectively. These results indicate that the classification prediction of GOS score using the hybrid CNN-SVM model is optimal.

**Table 4:** Accuracy and area under the curve of the four models.

Model	ACC^a^, %		AUC^b^, %	
	Training set	Testing set	Training set	Testing set
BP^c^	83.63	76.50	86.57	78.93
CNN^d^	87.63	84.00	87.18	84.21
SVM^e^	90.13	87.50	91.24	88.35
CNN-SVM	94.13	93.00	96.89	94.68

^a^ACC: accuracy.

^b^AUC: area under the curve.

^c^BP: back propagation.

^d^CNN: convolutional neural network.

^e^SVR: support vector machine.

**Figure 5:**
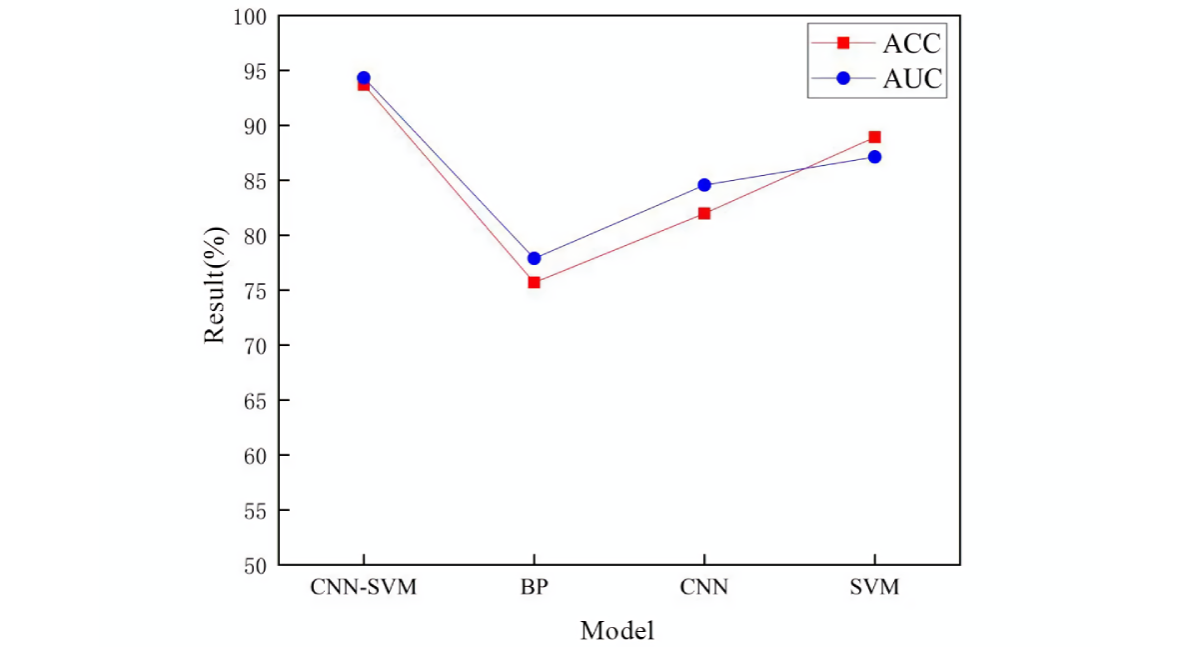
Accuracy and area under the curve of the 4 models in the external validation set. ACC: accuracy; AUC: area under the curve; CNN-SVM: convolutional neural network–support vector machine; BP: back propagation; CNN: convolutional neural network; SVM: support vector machine.

To further validate the reliability and merit of CNN-SVM, 111 data records from the First Affiliated Hospital of Anhui Medical University were used for validation. Eleven types of input variables collected were fed into the 4 models to compare the classification results; the experimental results are shown in Figure 5. The accuracy of GOS score classified by the 4 models (CNN-SVM, BP, CNN, and SVM) was 93.69%, 75.68%, 81.98%, and 88.29%, respectively, and the AUC was 94.32%, 77.89%, 84.57%, and 87.12%, respectively. The accuracy and AUC of CNN-SVM model were the best. Therefore, the CNN-SVM model is still the optimal model for predicting the GOS classification model through the validation of external hospital data.

### Predicting Length of Hospital Stay in Patients With TBI

To establish the optimal model for predicting the length of hospital stay for patients with TBI, this study compared the construction of CNN, SVR, and BP neural network models based on the design of a CNN-SVR model. The *R*^2^ and MAPE between the 4 models (CNN-SVR, BP, CNN, and SVR) for predicting length of stay and the true length of stay in the training and test sets are presented in Table 5. The CNN-SVR hybrid model exhibited the smallest error in the prediction results. Compared with that of the BP, CNN, and SVR models, MAPE was reduced by 7.61%, 10.15%, and 3.65%, respectively, indicating that the hybrid CNN-SVR model optimally predicted the length of hospital stay with higher prediction accuracy. The *R*^2^ of the CNN-SVR model was higher than that of the other 3 models, with values of 0.96 and 0.93 for the training and test sets, respectively, indicating that the CNN-SVR had the best fit.

These results indicated that the CNN-SVR model had high regression fit and regression accuracy for the training samples and good learning ability. The model could be trained to the maximum extent with the existing data while accurately approximating the actual values of the training samples. In general, the regression fit and prediction accuracy of the model for the predicted samples were lower than that of the modeled samples. A smaller difference between these parameters indicated better generalization ability of the model. The evaluation data of the models revealed that the CNN-SVR model most closely approximated the actual prediction ability and modeling effects with the smallest difference. This indicated better robustness and actual generalization performance of the CNN model and that this model was most suitable as the prediction model.

**Table 5:** Mean absolute percentage error and coefficient of determination for four model predictions.

Model	MAPE^a^, %			*R* ^2^	
	Training error	Testing error	Training error		Testing error
BP^b^	13.18	18.33	0.82		0.79
CNN^c^	15.29	20.69	0.76		0.73
SVR^d^	10.86	14.37	0.89		0.85
CNN-SVR	8.12	10.72	0.96		0.93

^a^MAPE: mean absolute percentage error.

^b^BP: back propagation.

^b^CNN: convolutional neural network.

^b^SVR: support vector regression.

**Figure 6:**
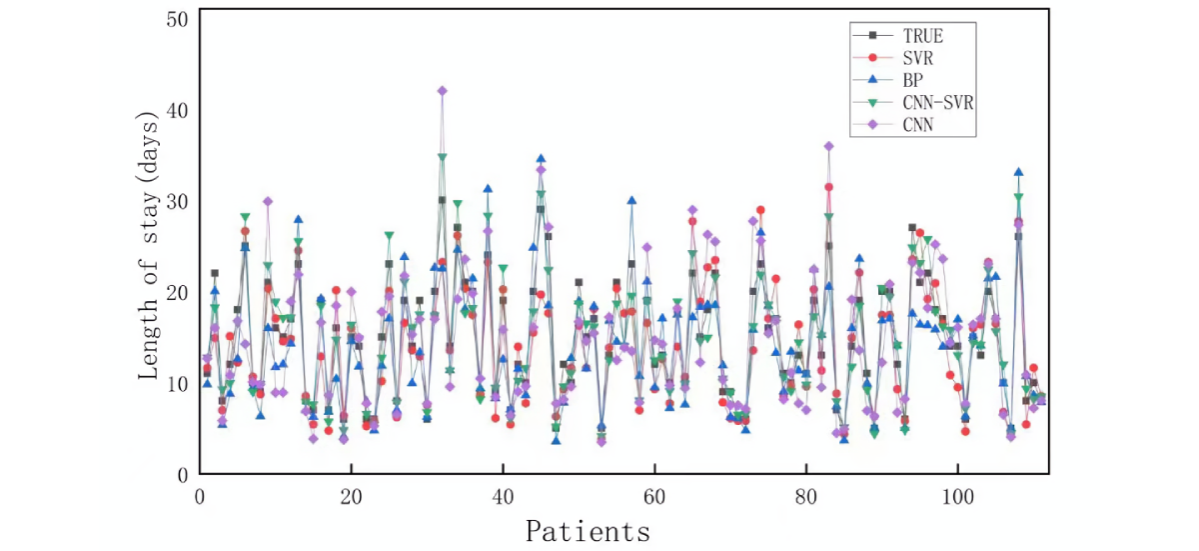
Comparison of the predicted results of the 4 models with the true values. SVR: support vector regression; BP: back propagation; CNN-SVR: convolutional neural network–support vector regression; CNN: convolutional neural network.

To further compare the reliability of the algorithms, 111 data records from the First Affiliated Hospital of Anhui Medical University were used for external validation. In total, 11 types of input variables from the collected data were input into the 4 models to compare the strengths and weaknesses of the models’ prediction effects. The experimental results are presented in Figure 6. Overall, the CNN-SVR predicted hospital length-of-stay contours were generally consistent with the patients’ true length-of-stay contours. And we further calculated the MAPE and *R*^2^ for CNN-SVR, BP, CNN, and SVR models as 10.44%, 17.60%, 20.71%, and 16.00%, and 0.92, 0.76, 0.73, and 0.79, respectively. The CNN-SVR model performed better with regard to both MAPE and *R*^2^ metrics. Therefore, the CNN-SVR model was the optimal model for predicting the length of stay of patients with TBI, as validated by external hospital data.

## Discussion

### Principal Findings

It is feasible to apply CNN, SVM, and SVR to the development of hybrid prediction models, which outperform traditional algorithms. In this study, we compared and constructed 4 models for each of the 2 prediction results, and the 2 hybrid models, CNN-SVM and CNN-SVR, performed the best in all metrics in the prediction results. The first was a hybrid CNN-SVM model for predicting GOS scores, combining the respective strengths of CNN and SVM, with accuracies of 94.13%, 93.00%, and 93.69% and AUCs of 96.89%, 94.68%, and 94.32% in the training, testing, and external validation sets, respectively. The second model was a hybrid CNN-SVR model for predicting actual length of stay with MAPE and *R*^2^ of 8.12% and 0.96, 10.72% and 0.93, and 10.44% and 0.92 in the training, test, and external validation sets, respectively. The data were optimal, indicating that our prediction model has high reliability and the results hold clinical utility. To our knowledge, this is the first study to build hybrid prediction models based on clinical data for prognosis and length of stay in hospital for patients with TBI.

### Comparison With Prior Work

Previous studies have proposed a linear regression (LR) scoring system for clinical studies, but its specificity and sensitivity are low, and its predictive performance is inferior to that of multivariate prediction models. Moreover, when LR is applied to describe complex multivariate nonlinear relationships, it may have low robustness and often requires complex transformations due to multicollinearity between variables [22]. Our machine learning models represent a new generation of multivariate statistical methods that can deal more effectively with multidimensional factors and are suitable for incorporating a wider range of risk factors for prediction. This ability reduces the reliance on practitioner experience and ensures objective results. For example, prediction of delayed graft function after kidney transplantation revealed that SVM-based machine learning exhibited better performance compared to LR [23]. Indeed, Feng et al [24] compared 22 machine learning methods with LR and reported that the AUC of LR was 0.83 with an accuracy of 88%, while almost all machine learning algorithms achieved higher AUC than that of LR. Compared to traditional methods, machine learning methods offer advantages in feature selection; the more factors considered, the more accurate the predictions become. In fact, machine learning methods have been applied to the field of TBI [9,25-27] using classical algorithms like artificial neural networks and BP neural networks, but these algorithms have serious shortcomings. This has also inspired us to apply the next generation of machine learning methods to clinical applications.

We used CNN in our study because it is a powerful machine learning model commonly used in the field of neurosurgery to analyze cranial CT scans [28-30]. With the development of computational power, the network depth of CNNs is increasing, enabling more accurate approximations of nonlinearly increasing objective functions. However, this is accompanied by increasing complexity of networks, making them difficult to optimize and prone to overfitting. Therefore, we introduced SVM and combined the two to build a more reliable model for a classification problem like predicting a patient’s prognosis (GOS score); on the contrary, a patient’s length of stay constitutes a regression prediction problem and SVR, a branch of SVM, was proposed precisely to solve the regression problem; therefore, we combined SVR and CNN to build a hybrid model for predicting the length of stay. The comparative validation shows that our model outperformed CNN, SVM or SVR, BP models built separately in training, testing and external validation sets. This confirms our conjecture that it is feasible and effective to use novel machine learning methods and combine their respective strengths in building hybrid models to solve prediction problems, and the results are clinically relevant.

Patients with TBI are generally sicker and have longer hospital stays than other patients, and this new form of hybrid predictive model could provide a reliable reference for health care decision makers in their work and help in managing patients more accurately. The 11 categories of input variables are available in previous studies, and these are all commonly used clinical data in the field of TBI that are easy to collect, which also demonstrates the operational and practical nature of our study.

### Limitations

Although our study lays the groundwork for the use of machine learning–based modeling in the field of TBI, several limitations should be acknowledged. First, machine learning methods are a computational construct unfamiliar to most physicians and may be dismissed as esoteric or unproven. However, with the rapid advancement of technology, artificial intelligence and machine learning will inevitable become widely used tools in the future. Second, due to the location of the Children’s Hospital of Anhui Province in our area, the sample size for severe TBI in children may be insufficient. In this regard, it may be necessary to cooperate with the children’s hospital at later stages to collect data from as many children with TBI as possible to further refine the model. In this study, we collected patients’ past medical history and classified them into 6 categories, which may not have a large enough sample size, and there are other types of past history that may affect the length of stay; later studies need to further expand the sample size to improve the accuracy of the model. In addition, there may also be small significant relationships between the input data; for example, a patient’s GOS score on admission and a head CT suggestive of brain herniation may have a significant impact on the model. This involves multicollinearity between input variables and feature selection, which is the focus of our next phase of research.

There has been an increasing number of recent reports on the detection of body fluid markers in patients to predict patient prognosis [31,32]. Although prediction performance in these studies did not supersede that of machine learning–based models, these studies have provided insight with regard to collection of relevant predictors as input data for machine learning models. Despite the limitations of this study, it is the first to use a next generation algorithm to build hybrid models to predict prognosis and length of stay in hospital for patients with TBI, and our models work better compared with traditional algorithms, demonstrating that CNN-SVM and CNN-SVR models can be useful in clinical work.

### Conclusions

In summary, our study is the first to combine multiple novel machine learning methods to develop hybrid models for application to TBI. Our hybrid models achieved excellent results and predicted target values quickly and accurately, with more stable performance. Further replication of the model may enable clinical teams and hospital managers to work collaboratively to provide optimal clinical care and may assist inexperienced practitioners in small remote or rudimentary facilities. We believe that our approach will provide more robust and accurate predictions and these can be updated in real time, with crucial implications for clinical work.

## Abbreviations

AUC: area under the curve
BP: back propagation
CNN: convolutional neural network
CT: computed tomography
GCS: Glasgow Coma Scale
GOS: Glasgow Outcome Scale
ICU: intensive care unit
LR: linear regression
MAPE: mean absolute percentage error
SVM: support vector machine
SVR: support vector regression
TBI: traumatic brain injury
